# Supplementary material for: Splice-Junction-Based Mapping of Alternative Isoforms in the Human Proteome
Source: Cell Rep. Author manuscript; Available in PMC 2020 Jan 15. (PMC6961840; doi:10.1016/j.celrep.2019.11.026)
Supplement: 2 [file NIHMS1546469-supplement-2.zip › DF1/PXD006675/RightAtrium/RightAtrium_55_PHPT1_IHVYGYSMQMRPTCVPLGASG.pdf]

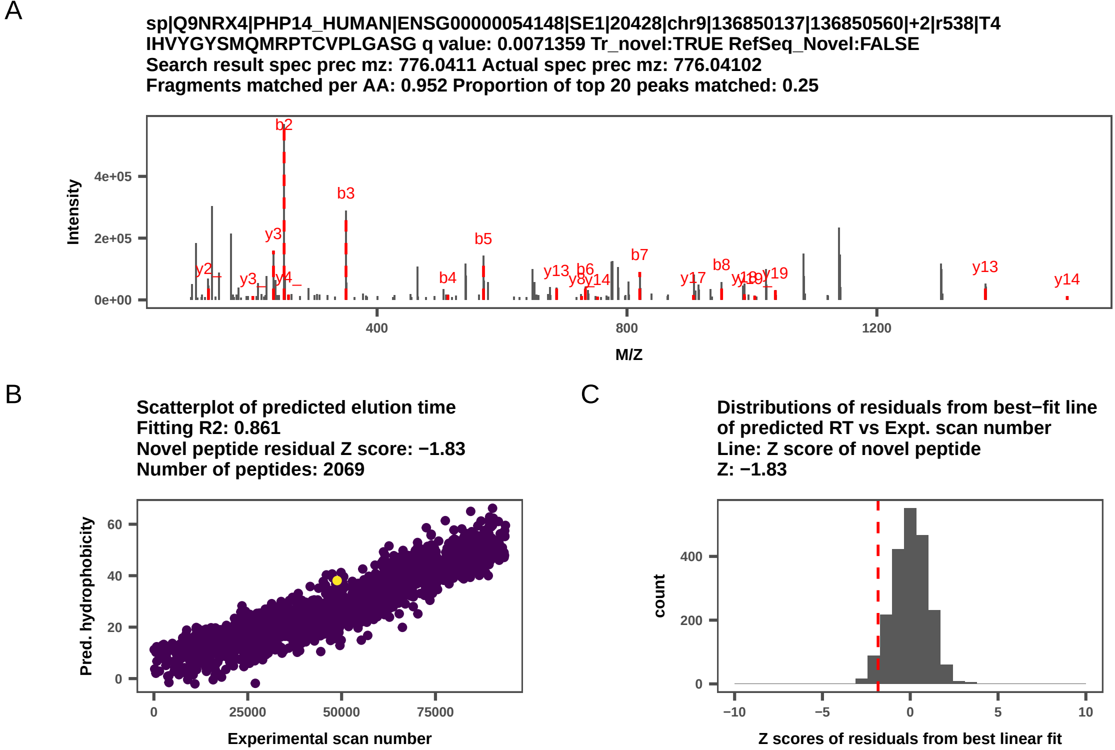
A
sp|Q9NRX4|PHP14_HUMAN|ENSG00000054148|SE1|20428|chr9|136850137|136850560|+2|r538|T4
IHVYGYSMQMRPTCVPLGASG q value: 0.0071359 Tr_novel:TRUE RefSeq_Novel:FALSE
Search result spec prec mz: 776.0411 Actual spec prec mz: 776.04102
Fragments matched per AA: 0.952 Proportion of top 20 peaks matched: 0.25
Intensity
4e+05
2e+05
0e+00
b2
b3
y3
y2
y3
y4
b4
b5
y13
b6
y8
y14
b7
y17
b8
y18
y19
y13
y14
400
800
1200
M/Z
B
Scatterplot of predicted elution time
Fitting R2: 0.861
Novel peptide residual Z score: −1.83
Number of peptides: 2069
Pred. hydrophobicity
60
40
20
0
0
25000
50000
75000
Experimental scan number
C
Distributions of residuals from best−fit line
of predicted RT vs Expt. scan number
Line: Z score of novel peptide
Z: −1.83
count
400
200
0
−10
−5
0
5
10
Z scores of residuals from best linear fit
